# Challenges and possibilities when doing research on active school travel interventions in a school setting - a non-randomized pilot study assessing feasibility of an evaluation design

**DOI:** 10.1186/s12889-025-21445-9

**Published:** 2025-01-23

**Authors:** Mathias Andersson, Elena Tseli, Anna-Karin Lindqvist, Stina Rutberg, Annie Palstam

**Affiliations:** 1 School of Health and Welfare, Department of Medical Sciences, Dalarna University, Falun, Sweden; 2 Department of Neurobiology, Care Sciences and Society, Division of Physiotherapy, Karolinska Institutet, Huddinge, Sweden; 3 Department of Health, Education and Technology, Luleå University of Technology, Luleå, Sweden; 4 Sahlgrenska Academy, Institute of Neuroscience and Physiology, University of Gothenburg, Gothenburg, Sweden; 5 Department of Rehabilitation Medicine, Sahlgrenska University Hospital, Gothenburg, Sweden; 6 School of Health and Walfare, Dalarna University, Falun, 791 88 Sweden

**Keywords:** Active transport, Commuting, Children, Measurement methods

## Abstract

**Background:**

A wide range of school interventions have been launched to increase childrens’ physical activity. Evaluation of the effectiveness of interventions requires suitable study designs and feasible quantitative evaluations relating to the school setting. The purpose of this study was to assess the evaluation design and methods for data collection, in order to make decisions about approaching forthcoming studies of the effectiveness of active school travel (AST) interventions.

**Methods:**

Children from four Swedish schools in fifth grade (11–12 years old) participated in this non-randomized pilot study, two schools received an AST intervention and two schools were controls. The school-based AST intervention Sustainable Innovation for Children Transporting Actively (SICTA) was conducted by teachers in the classroom setting during four weeks. To assess feasibility of the evaluation design and methods for data collection a combination of quantitative and qualitative methods were applied, using participation- and response rates, a feasibility questionnaire and focus group interviews.

**Results:**

Out of 25 potential schools, four schools accepted participation with explicit allocation requests preventing randomization. Out of 181 children, 107 children (59%) accepted participation. A total of 82% of the participating children reported active travel before the AST intervention, and 80% found reporting of daily school travels in the web-based survey to be easy. The children were in general positive about participating in the study and the methods for data collection were considered easy for the participating children to conduct and to blend well with usual school activities. There was an imbalance in reporting rates between intervention and control schools as well as a decrease in reporting rates during the study period.

**Conclusions:**

Our results highlight the complexity and challenges in conducting controlled research among school children. Although children were positive about participation and found reporting to be easy, our results invoke the need to use alternative research designs and recruitment strategies that also attract children using non-active modes of travel when evaluating AST interventions in school contexts.

**Trial registration:**

The study is registered 2023/11/02 with Researchweb, the Swedish Register for Research with registration number 281,543. The registration can be reached via this link: https://www.researchweb.org/is/sverige/project/281543.

## Background

The prevalence of physical activity (PA) among children has declined remarkably during the last few decades [1, 2], even though daily PA is vital for both physical and psychological health, especially for children and adolescents [3]. A wide range of initiatives have been launched to increase the amount of PA during leisure time among children [4, 5], as well as during school hours [6]. Using schools as the setting for an intervention offers the opportunity to reach almost all children, which is a great advantage given the inequities in PA and associated aspects of health [7]. School interventions aimed at increasing PA often emphasize activities in classrooms and during school breaks [6] but can also focus on promoting active school travel (AST). Several initiatives, such as Walking School Buses [8, 9], Safe Routes to School [10, 11], and cycle training programs [12, 13], have been launched to promote AST. While these interventions show potential, their effectiveness varies, and challenges relating to evaluations and understanding of the complexity of these health-related interventions are crucial for determining their success [1, 14–16]. In addition to increased PA, AST may contribute to reduced traffic load around the school environment [17] and shows associations with reduced air pollution [18] and beneficial environmental impact [19]. Each of these outcomes represents one of the major relevant societal challenges to achieving the United Nations’ Sustainable Development Goals [20].

One example of a school-based intervention aimed at promoting AST was developed at Luleå University of Technology in 2016 [21, 22]. The intervention, “Sustainable Innovation for Children Transporting Actively” (SICTA), consists of weekly assignments for children to solve during AST. Subjective evidence, from qualitative evaluations of the SICTA intervention have shown that the intervention motivates and inspires children to use AST, and some children express having developed new habits of more regular active travels after the SICTA intervention [22–24]. However, quantitative evaluation of the effectiveness of the intervention requires suitable study design and feasible quantitative evaluation methods related to the interventions in a school setting.

Measures that could be used to monitor PA in school settings include accelerometers [25, 26], GPS trackers [27], and weekly questionnaires [26], but each of these entails some limitations in terms of practicality [26, 28] or validity [26]. Hence, we have recently developed a web-based survey (wASTapp), distributed daily via Short Message Service (SMS), in which children self-report travel mode, commuting time, and distance to assess daily AST [*in manuscript*]. The wASTapp is intended for quantitative evaluation of AST [29] but needs to be further tested in a real-life intervention context to evaluate its practicality and acceptability before an effectiveness study is performed. Also, psychosocial aspects have been shown to be positively related to AST, for example confidence in ability, attitudes, social support, and social norms which is why such aspects could be of interest as mediators for intervention outcomes [30]. Schools are complex environments [31], and SICTA is considered a complex intervention, requiring adaptive components and competence by those who deliver and/or receive the intervention [32]. Under such circumstances, the Medical Research Council (MRC) framework for the development and evaluation of complex interventions [32, 33] provides a supporting structure for planning and conducting studies to assess feasibility prior to large scale evaluations of effectiveness. The aim of this study was therefore to assess the feasibility of a planned evaluation design and methods for data collection to make decisions about approaching forthcoming studies of the effectiveness of the SICTA intervention.

## Methods

### Study design

In accordance with the conceptual framework of feasibility studies [34], a non-randomized controlled pilot study was carried out, applying a combination of quantitative and qualitative methods. The study evaluated feasibility outcomes in relation to the evaluation design and methods for data collection in preparation for future evaluations of AST interventions. The Swedish Ethical Review Authority approved the study (no. 2021–03783).

### Setting

The study was carried out during February–March 2022 in Falun, Sweden. Falun has a geographical area of 2275 km^2^ [35], and about 60,000 people live in the municipality, of whom about 39,500 live in the urban area [36]. The municipality has 25 elementary schools [37].

Falun is located in the province of Dalarna, in central Sweden, and experiences winter road conditions with snow and ice (December–April). During the beginning of the study period, the third wave of the COVID-19 pandemic swept over Sweden. This wave had implications for the study, which had to be conducted mostly through virtual interaction between the researchers and the children, parents, and teachers involved. However, focus group interviews succeeding the SICTA intervention were not affected by COVID-19-restrictions and therefore conducted onsite.

### Participants and recruitment

Children in fifth grade (age 11–12 years) were the target population. In collaboration with Falun municipality (the Child and Education Administration as well as the Sustainability Strategist), invitations to participate in the study were distributed to principals in all 25 public schools in Falun. Three principals expressed interest for their schools to participate, two with explicit requests to participate as intervention schools and one with explicit request to participate as a control school. After a second request from the Child and Education Administration, a fourth principal agreed for their school to participate in the study, as a control school. The four principals were further informed about the study and contacted their personnel. Neither participating schools nor children received any payment for their participation.

### Description of the SICTA intervention

The SICTA intervention was delivered in the classroom setting for 4 weeks, consisting of weekly assignments to promote AST. The assignments are related to the national curriculum, and teachers together with the children decided on relevant SICTA assignments for children to solve on the way to/from school, guided by an online platform (the teachers’ portal) [22].

For example, one assignment concerns parental fears and traffic safety when cycling or walking to school. The assignment is inspired by Astrid Lindgren’s book “Ronja Rövardotter” (Ronia, the Robber’s Daughter), which follows Ronja as she explores the forest and learns about its dangers. The book is a classic in Swedish literature, known for its themes of independence and bravery. The task addresses parents’ concerns about their children walking or cycling to school and provides an opportunity to discuss traffic safety.

In the first step, the children are asked to identify dangers on their way to school and document these dangers through photos or notes. Following this, the children read an excerpt from “Ronja Rövardotter” in the classroom where Ronja’s father warns her about the forest’s dangers. This is followed by discussions of the text in small groups.

Next, the children complete a writing assignment where they write a text mimicking Lindgren’s style about a child cycling or walking to school for the first time and their parents’ warnings. The children consider what dangers they might face and how to overcome them. They can also do sub-assignments to reflect on and discuss the dangers with their parents or interview an adult about their fears regarding the school route.

Lastly, the assignment is followed up by sharing and discussing the texts in small groups, focusing on common fears and traffic safety. The online platform includes several assignments for different subjects that the teacher could choose from, moreover, the teachers could also choose to develop their own assignments with inspiration from the existing ones.

The SICTA intervention consists of five steps: (1) Caregivers are informed during a parental meeting. (2) Children are involved and given information about the benefits of AST. (3) To promote AST, teachers and children decide on suitable assignments together. (4) Weekly assignments are performed during a 4-week period, while AST is measured and reported. (5) The SICTA intervention ends with an evaluation and a celebration.

### Control schools

The control schools did not receive any AST intervention (passive control).

### Outcome measures and data collection procedures

The outcome measures and data collection procedures are presented below in three parts. The first section describes the measures and procedures intended for describing the characteristics of the study sample. The second section describes the measures and procedures intended for evaluation of the SICTA intervention, and the third section details the targeted measures and procedures for the assessment of this study’s feasibility objectives.

### Measures to describe characteristics of the study sample

#### Health-related quality of life (HRQoL)

To describe the population, HRQoL was self-reported using a child friendly EQ-5D version (EQ-5D-Y) [38, 39]. The EQ-5D-Y can be summarized using an index value (1–3), where index 1 indicates very good HRQoL [38, 39]. Data collection took place one week before the intervention/control phase.

### Outcome measures for evaluation of the SICTA intervention

#### AST

In this study, AST was defined as actively traveling to school for the whole or part of the distance by walking, cycling, or school bus (children traveling by school bus also travel part of the distance actively [40, 41]). The wASTapp was used to report daily travel mode, commuting time, and distance traveled and was accessible from computers and smartphones. The children logged in via their digital school account using a personal “research ID.” As an option, a web link to the wASTapp was distributed daily via SMS to the child’s (or parents’) smartphone.

A paper report was also available for teachers to distribute to children if needed, as an analogue option for daily reports.

School travel was registered for each direction to and from school, with a maximum of 10 reported trips each week. Additionally, in two schools (one control and one intervention school) wASTapp reports were complemented with accelerometers (control school), and accelerometers together with GPS trackers (intervention school). Data collection included daily reports to and from school, one week before and one week after the intervention phase.

#### Mediators of AST

Three self-report questionnaires were used to collect data on social mediators of AST: Behavioural Regulation in Active Commuting to and from School questionnaire in Sweden (BRACS-SWE) [22, 42], Perceived Autonomy Support Scale for Active Commuting to and from School (PASS) [43], and Basic Psychological Need Satisfaction in Active Commuting to and from School (BPNS-ACS (SWE)) [44]. Furthermore, to collect data on parental attitudes about AST, caregivers completed the Parents Intentions to Let their Children use AST (PILCAST-52) questionnaire [45] via the web (data not reported in this study). Data were collected one week before and one week after the intervention phase.

### Measures and procedures to assess feasibility of the evaluation design

#### Participation and response rates

Objective measures used as a quantitative indicator to describe the feasibility of the study design and methods for data collection were based on following data sources: recruitment of schools, number of schools and children accepting participation in the study, reporting rates of the daily school travels in the wASTapp, response rates of the questionnaires (BRACS-SWE, PASS, BPNS-ACS (SWE), and EQ-5D-Y), and adherence rates for wearing measuring devices (accelerometer and GPS tracker).

#### Feasibility questionnaire

The research group created a 10-item questionnaire (“the feasibility questionnaire”) to assess indicators of feasibility [34] based on children’s opinions of the research evaluation process and methods for data collection. A four-point Likert scale was used in which 1 indicated a very positive and 4 a very negative response. Free-text comments in the feasibility questionnaire for additional clarification were optional. The feasibility questionnaire was distributed together with the abovementioned other three questionnaires one week after the intervention phase.

#### Focus groups

**Fig. 1:**
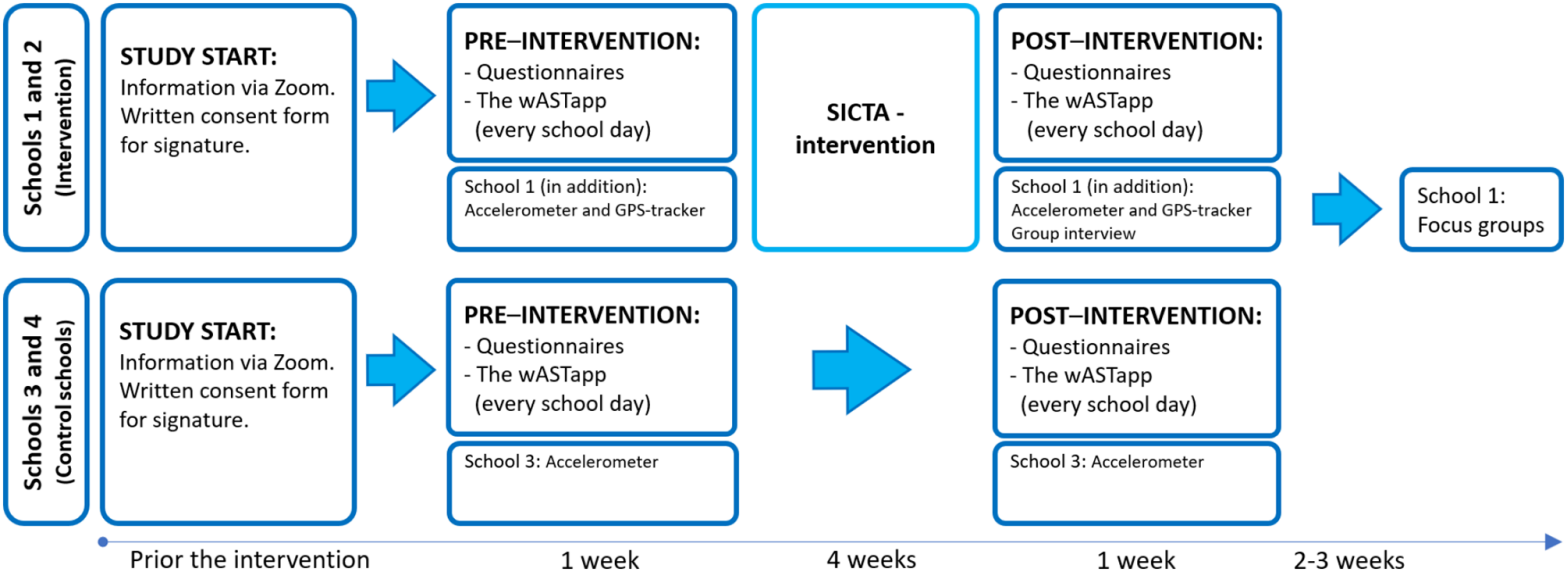
Timeline and content of the study procedure

**Fig. 2:**
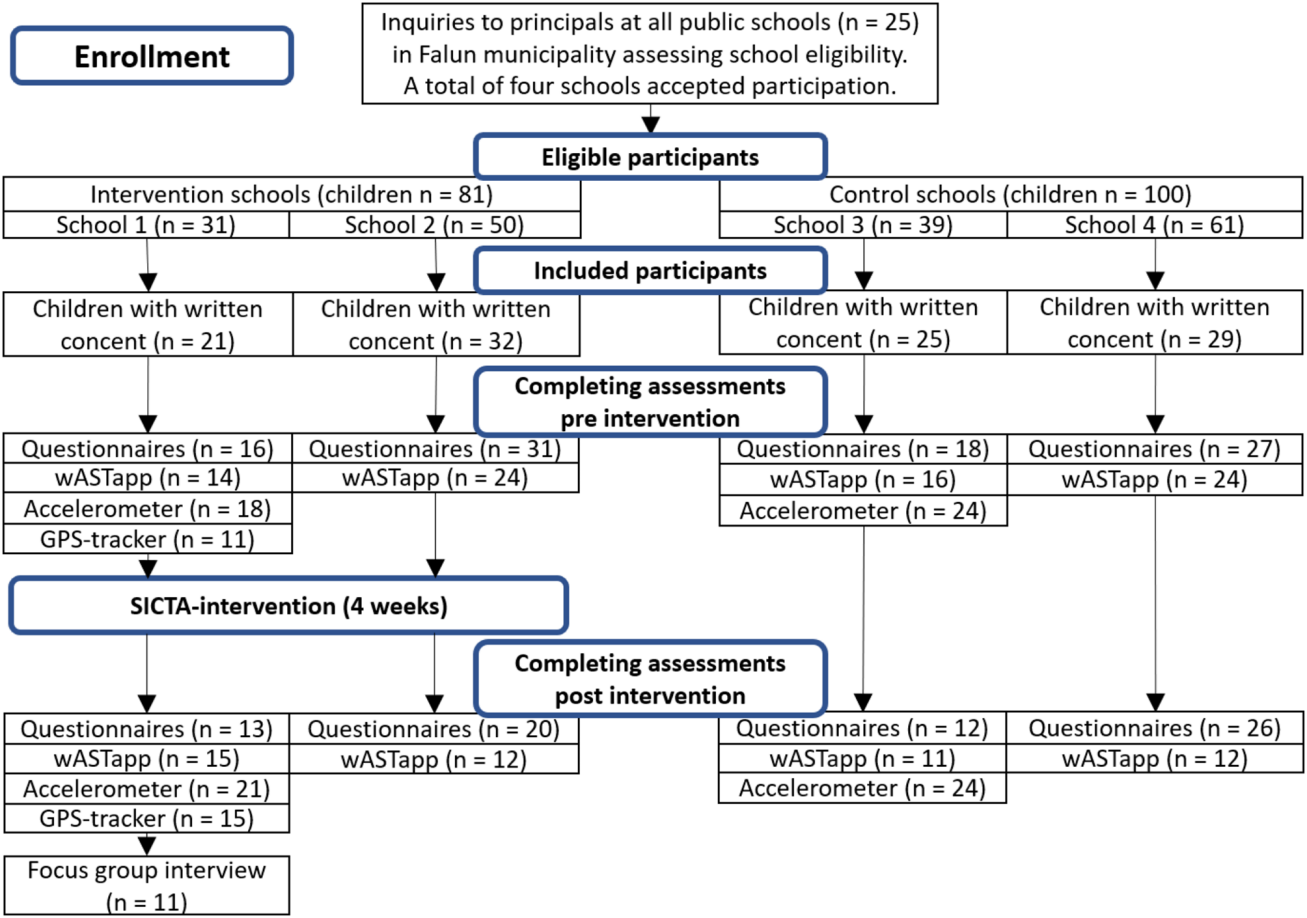
Flowchart of enrollment and inclusion of study participants in intervention and control schools, and children completing assessment pre- and post-intervention. Clarification: Any reports in the questionnaires and wASTapp were counted as a valid result

Semi-structured focus group interviews [46] were performed to explore the children’s experiences with the research and methods for data collection and to gain a richer understanding of aspects of feasibility from their perspective. All children who participated in the study at one intervention school (where all methods for data collection were included) were invited to participate in the focus groups (Figs. 1 and 2). The focus groups were conducted according to a semi-structured interview guide based on the feasibility questionnaire. One of the authors (MA) moderated all focus groups with the children. To ensure dependability, the interview guide was followed carefully, and probes were used to facilitate discussions. The focus groups were carried out after school in the classroom, and the children could sign up to one of three different occasions, two or three weeks after the SICTA intervention.

### Study procedure and data analysis

#### Study procedure

Information about the study was given to teachers, children, and caregivers using video conference. Teachers were oriented first, followed by the children. During the introduction, the children were informed about the study and asked to obtain signed informed consent from their caregivers to participate. Children were informed that they were part of a research project to test research methods related to assessing school travels. Additionally, children in ools were informed about the SICTA intervention.

Caregivers were informed in a parents’ evening presentation as well as by printed study information, with recorded information videos and text made available on the study website. In case of two caregivers, both were asked to sign informed consent for their child. For all families, it was emphasized that participation was voluntary and could be discontinued at any point without giving reasons. For ethical reasons, all fifth-grade children from the participating schools could fill in the questionnaires and use the daily wASTapp, but data were collected only for children with a signed informed consent obtained from a parent and/or legal guardian. For the same reason and to avoid non-equal situations, all children in the intervention schools participated in the previously described SICTA-intervention.

After the introduction to the study process, individual coded folders for each child were provided to the teachers, who distributed the materials to all children one week before the intervention. The folders included questionnaires and a “junior researcher business card” (with an individual ID number, the study website address, and password to the wASTapp). Via video conference, the researchers (MA and AP) gave instructions to all fifth-grade children and their teachers about how to use the password for login and how to register travel mode, commute time, and distance (using Google Maps™) in the wASTapp.

General information about the study, instructions related to wASTapp, and how to measure time and distance were also presented on the study website (https://www.du.se/aktivaskoltransporter) in the form of short instructional videos, as well as texts.

Instructions on use of accelerometers and GPS trackers were provided, where these were distributed. Following the SICTA intervention, the feasibility questionnaire and focus group interviews were added to assess aspects of feasibility. The timeline of the study procedure is further detailed in Fig. 1.

### Data analysis

#### Participation and response rates

Quantitative data were analyzed using the Statistical Package Software for the Social Sciences (SPSS version 28.0.1.0, Chicago, IL, USA). Descriptive data are presented as median and min–max or interquartile range (IQR) for ordinal and continuous variables or as number (n) and percentage (%) for categorical variables. For comparison between groups on ordinal variables, the Mann–Whitney U test [47] was used. In the analyses, a p-value < 0.05 was considered significant.

#### Feasibility questionnaire

The ratings from the feasibility questionnaire were dichotomized by answer choices that were positive (1: Very fun/ Very well/Very easy; 2: Fun/Well/Easy) and negative (3: Not fun/Not well/Not easy; 4: Boring/Not at all well/Not at all easy). Ratings were reported as proportions of positive and negative responses.

#### Focus groups

Qualitative data were analyzed using inductive manifest content analysis inspired by Graneheim and Lundman for content analysis [48]. MA transcribed the interviews. The transcriptions were then read separately by the two authors MA and AP, one being a junior researcher (MA) and the other a senior researcher with many years of experience in qualitative research (AP). The transcribed interviews were read several times to obtain a sense of the overall data, identified meaning units, and individually assigned codes to the meaning units, as suggested by Graneheim and Lundman [48–49]. The two authors then compared their coding for discrepancies, discussing them several times until consensus was reached [48]. The codes were then collated into preliminary categories by MA, with the aim of staying close to the text [49]. A third author (SR), a senior researcher with many years’ experiences in qualitative research, was involved to discuss and validate the preliminary categories, and after discussions among the three authors, the preliminary categories were collated into final categories. Quotes were used to strengthen the credibility of the analysis [48–49].

## Results

### Participants

Of 25 potential schools informed about the possibility to participate, principals from two schools expressed interest and agreed to participate if allocated to intervention group. One school agreed to participate as control and after further invitations, an additional school agreed to sign up to be included as a control school so that overall, four schools participated: two as intervention schools (*n* = 50 and *n* = 31 children, from each school respectively), and two as control schools (*n* = 61 and *n* = 39 children, from each school respectively). The schools had 181 eligible fifth-grade children in total, of whom 107 (59%) accepted participation with written consent, evenly distributed in the intervention and control schools (Fig. 2), with 60 girls and 47 boys. The children had a median reported HRQoL of 1 (1–3). A total of 82% of reported trips involved active travel, and the median distance to school was 1400 m. Characteristics of the study population are presented in Table 1.

**Table 1:** Characteristics of the study population

	Total *n* = 107	Intervention schools, *n* = 53	Control schools, *n* = 54
Female, n (%)	60 (56.1)	30 (56.6)	30 (55.6)
HRQoL (EQ-5D-Y index), median (min–max) 1 = best health imaginable to 3 = worst health imaginable	1 (1.0–1.8)	1 (1.0–1.8)	1 (1.0–1.8)
Distance to school in meters, median (IQR)	1400 (704–2020)	1600 (825–2000)	1180 (609–2385)
Active school travel (walk, bicycle, bus), n (%)	387 (81.6)	213 (91.4)	174 (72.2)
Travel mode to/from school, n (%)			
- Walk	319 (67.3)	193 (82.8)	126 (52.3)
- Bicycle	11 (2.3)	8 (3.4)	3 (1.2)
- Car	87 (18.3)	20 (8.6)	67 (27.8)
- Bus	57 (12.0)	12 (5.1)	45 (18.7)

Missing data: distance to school, *n* = 30; travel mode to/from school, *n* = 593 (of 107 × 10 possible trips); EQ-5D-Y: intervention schools, *n* = 9 and control schools, *n* = 8

Abbreviations: HRQoL, health-related quality of life; IQR, interquartile range

### Pre- and post-intervention questionnaire response rates

Before the intervention, 47 (89%) children from the intervention and 45 (83%) from the control schools answered the questionnaires (partially or completely), and 18 (86%) from one of the intervention schools and 24 (96%) children from one of the control schools wore accelerometers (during one or several days) (Fig. 2). After the intervention, 33 (62%) children from the intervention and 38 (70%) from the control schools answered the questionnaires (partially or completely), and 21 (100%) children from one of the intervention schools and 24 (96%) children from one of the control schools wore accelerometers (during one or several days) (Fig. 2).

### Pre- and post-intervention wASTapp reporting rate

**Fig. 3:**
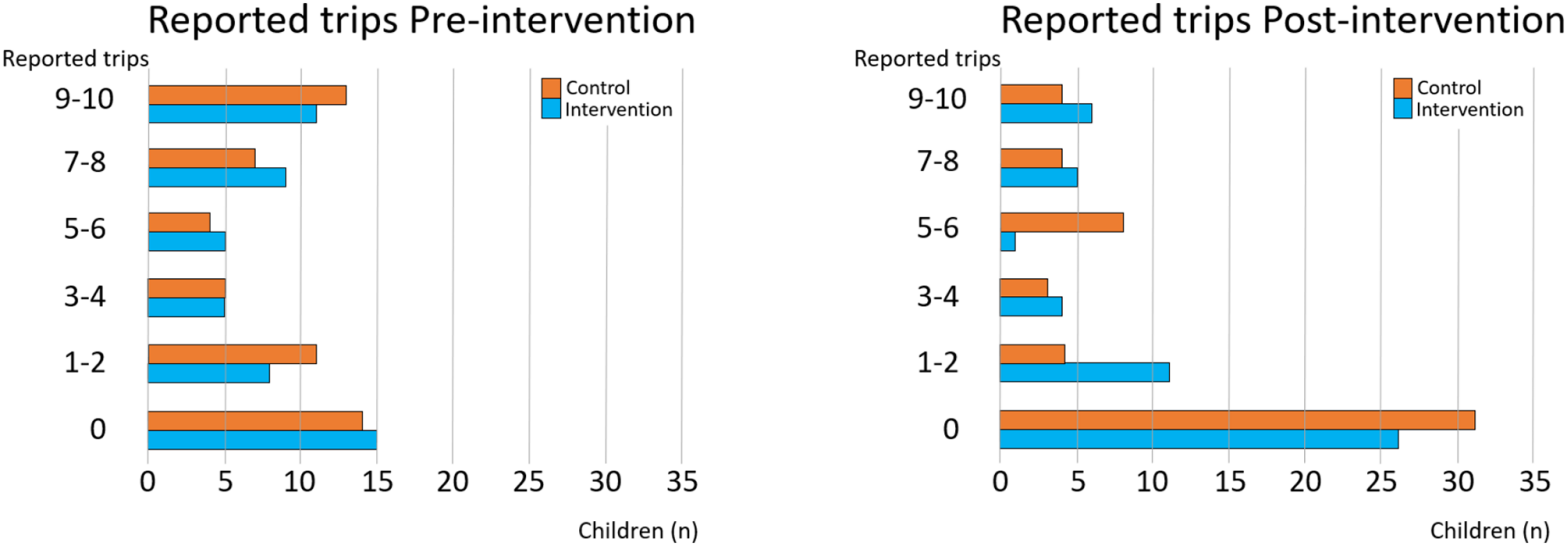
The number of reported trips in the wASTapp pre- and post-intervention

The number of reports in the wASTapp did not differ significantly between the intervention and control schools pre-intervention (*p* = 0.85) or post-intervention (*p* = 0.59). Before the intervention, intervention schools reported a median of three trips (IQR, 0–8), compared with a median of four (IQR 0–8) in control schools. After the intervention, intervention schools reported a median of one trip (IQR 0–4) and control schools a median of 0 trips (IQR 0–6) were reported (Fig. 3). No child chose to report using the paper version of the wASTapp.

### Feasibility questionnaire results

**Fig. 4:**
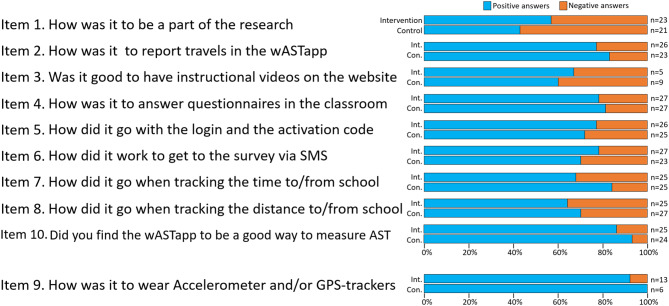
Questionnaire about the intervention. Gradings on item 1: 1 = Very fun; 2 = Fun; 3 = Not fun; 4 = Boring. Grading on items 2 and 4–9: 1 = Very well/Very easy; 2 = Well/Easy; 3 = Not well/Not easy; 4 = Not at all well/Not at all easy. Gradings on items 3 and 10: 1 = Yes; 2 = No; 3 = I did not visit the website. Positive answers in blue = Very fun/Very well/Very easy or Fun/Well/Easy, and negative answers in orange = Not fun/Not well/Not easy or Boring/Not at all well/Not at all easy. As noted in the text, answers were dichotomized into positive or negative responses. Item 9 was presented only to the two schools relevant for wearing the measurement equipment and is therefore displayed separately

In total, 58% of the children in intervention schools and 42% of children in control schools perceived the participation in the study as positive (item 1) (Fig. 4). Some children in control schools elaborated in free-text comments on their perception of reporting as being very boring. Four out of five children (80%) reported that the wASTapp was easy to use (item 2), and three out of four (74%) reported not using the resources with instructional videos describing how to report school travels in the wASTapp (item 3), although some children (one out of six) (17%) reported that the instruction videos on the website were useful (Fig. 4). Free-text comments also revealed that the children largely did not use the instructional videos, but one child wrote, “I don’t need them but others might.”

Four out of five children (80%) found the process of completing questionnaires in the clasroom to work well (item 4) (Fig. 4). In free-text comments, however, some children stated it to be tedious to have to respond to several questions that they perceived to be very similar: “Too many questions, and they were kind of alike.”

Three out of four (75%) children reported the wASTapp login on home computers or smartphones (item 5) to work well (Fig. 4), and free-text comments clarified that the login worked best on the device where the first login was performed: “It did not work on smartphone, but worked in school.” The option with a web link to the wASTapp distributed via SMS (item 6) worked well for three out of four children (75%), and once they logged in, most children reported that the measuring of time (75%; item 7) and distance (item 8; 68%) was easy (Fig. 4).

Children who wore GPS trackers and/or accelerometers received an additional question (item 9) about how it was to wear the devices during the week (data not included in Fig. 4). Although 95% of the children reported that the devices were easy to carry, some stated that the measuring devices were uncomfortable to wear: “It hurts and is boring.”

### Focus group results

Three focus groups were conducted with 11 children from the intervention school, mentioned above. Seven girls and four boys participated in the interviews, lasting 15–25 min each. Analysis of the focus group material resulted in three categories: *Participation was natural and acceptable*, *the practicality of the methods for data collection varied*, and *a variety of problem-solving strategies was used in order to contribute*.

### Participation was natural and acceptable

Most of the children did not think much about taking part in the research project and did not reflect on whether the research project resulted in any positive effects, as they found it to be a natural and acceptable part of their daily lives:

- *I didn’t think much about it* (participating in the research).
- *No*,* it was just like a normal day in school.*
- *It was not much different than what we use to do.*

However, some children noted that there were more children walking and cycling to/from school since the start of the research project, and some children stated that they found it fun to measure their distances to/from school and to see the collective distance when adding the walking and cycling measurements together in class. Furthermore, the children used descriptions that revealed an awareness of children of today as being generally sedentary, and they understood that the desirable behavior within the research project was to increase the amount of walking and cycling.

### The practicality of the methods for data collection varied

The children had different perceptions of the study’s methods for data collection, with several of them commenting on the questionnaires as being very comprehensive and their difficulty in understanding the purpose of all the questions. Others noted that the questions seemed to belong within specific categories and were therefore easy to answer. Some children found the measuring devices uncomfortable to wear, but others stated that they barely noticed wearing the devices to/from school.

Some children experienced problems when measuring the distance to/from school, but others found it to be quite simple. Most children found the wASTapp to be intuitive and easy to use for reporting their daily school travels:

- *Most of it was easy.*
- *And we measured distance in the classroom* (as a part of the education).
- *Yes*,* everything* (about the wASTapp) *was quite simple.*

Some children described having problems logging into the wASTapp, but others were able to log in without any difficulties even with logging in from different devices. Because of the restrictions of the pandemic, several children described being absent from school during parts of the project period. Their absences affected how much they had been able to participate and report, and they also indicated that their absence made it difficult to express thoughts on their participation.

### A variety of problem-solving strategies were used in order to contribute to the research

The children described different ways of coping with and finding strategies to solve potential problems and difficulties with contributing data to the research project. Some children with login problems either chose to circumvent the difficulties by using their private Google login, by searching for information on the project’s website, or by peeking at other children to see how they solved their login:

- *It worked quite well for me* (with the login), *I got in directly.*
- *But the first week I tried to change to the school account on my smartphone*,* but it did not work… but it worked well in school.*
- *I tried to register with my own account at home*,* but it did not work*,* and then I tried to change*,* but it did not work.*

Some children chose to ask the teacher for help with the login procedure or other practical aspects such as measuring the distance between home and school. Some also described using teacher support to understand items in the questionnaires, but the children implied that they did not ask their classmates for help.

Several children stated that they had difficulty remembering to report in the wASTapp each day. Some children expressed that they would have benefited from more support with reminders to report data during the week. They also suggested to include the option to report in the wASTapp for the previous day in case they forgot. Furthermore, some children noted that the GPS battery was running low and chose to solve the problem by charging the GPS tracker to assist with data collection during the week. At the same time, many children also stated that they chose to skip parts of the research (or reporting) where they encountered difficulties.

## Discussion

The aim of this study was to assess the feasibility of a planned evaluation design and methods for data collection to make decisions about how to proceed in forthcoming studies of the effectiveness of AST interventions.

### Evaluation of recruitment procedure and participation

Our results show that of 25 eligible schools, only four accepted participation. Moreover, at two schools, the principals expressed an interest in participation but with explicit requests to be in the SICTA intervention, and the principals at the other two schools agreed to participate as control schools. Allocation based on randomization thus was not feasible, in line with previous findings of challenges with the procedure for randomized controlled trials in school settings [50].

Of all eligible children, 59% agreed to participate with written consent, in line with results from studies using active consent procedures [51]. However, in contrast to reports on declining levels of AST [1], our results show that 82% of the participating children reported AST before the intervention, distributed between 91% of the children in the intervention schools and 72% in the control schools. This percentage of active travelers is remarkably high compared with previous findings [52], and it may be that children already using AST were more prone to participate in this study, as well as more willing to report their active school travels.

Furthermore, childrens reportings of satisfaction with participation was quite neutral, with children in intervention schools tending to report participation as more positive than children in control schools. Also, lower reporting rates were found among children in control schools, with some children expressing a negative experience of participating and of the study as being very boring. Other intervention studies in the school context also have identified difficulties with evaluation design using control groups and a randomized control design [50]. Still, schools are considered to be complex environments, and Medical Research Council guidelines recommend that many questions should be answered at different phases in the research process [32]. Hence, with the complexity arising from both the intervention’s components and from its interaction with the context of its implementation, further studies are needed addressing alternative designs for how to best evaluate AST interventions in a school context.

### Evaluation of data collection procedures

Overall, the children reported the practicality of the measurement methods as predominantly positive. Before the intervention, the children reported a median of 3–4 trips in the wASTapp. After the intervention, the report rate was lower, with a median of 0–1 reported trips. The wASTapp is based on reports directly from the child (first-hand source) in temporal proximity to the trips, resulting in reduced risk of recall or information bias [53]. Most children reported that the wASTapp was easy to use once they were logged in; however, the login process seemed to be an obstacle to reporting, and the children described using different strategies when sometimes struggling to report their travels. This issue indicates a crucial need to make investments in adjusting measurement methods to children in AST evaluations.

Accelerometers and GPS trackers are commonly used for objective measurements of PA, but these devices are considered difficult to administer to a large-scale population because they are labor intensive and costly [26]. In addition, cycling in particular is difficult to assess with these devices [54], an especially important issue in northern Europe, where cycling is common [55]. The advantages of the digitally distributed wASTapp include the ability to capture different travel modes in large populations simultaneously, without additional manual handling by researchers. However, there were some missing reports in the wASTapp, and it is not possible to determine if the missing data were related to absence because of the pandemic or to other aspects of feasibility. Self-reported measurement methods, such as the wASTapp, nevertheless could be used to investigate children’s AST behaviors rather than using objective measures of PA, given the sensitivity for measuring different modes of travel and that the daily reporting does not involve a risk of recall bias [56].

The pandemic precluded the intended data collection procedure, which subsequently was altered from an attended presentation in the classroom to a video conference presentation. This adjustment, together with consequences related to the pandemic, such as restrictions and sickness-related absences, might explain participation and reporting rates.

### Study limitations

One study limitation concerns the recruitment of schools, including the explicit conditions for participation by principals, which made a plan for randomization impossible. Also, the current evaluation design may have attracted children already using active methods for traveling to school, which would imply a recruitment bias. These issues pose obvious difficulties when designing a large-scale evaluation for intervention effects. Furthermore, feasibility results may be interpreted with the understanding that the study was conducted during the third pandemic wave, which may have interfered with aspects of feasibility concerning participation- as well as reporting rates.

### Suggestions for further research

The difficulties with allocation of schools based on randomization, together with participational dissatisfaction among children in control schools, invoke the need for exploring alternative study designs for large-scale evaluations. An example could be a quasi-experimental age-cohort design [57], which offers the advantage of not involving control groups. In making such a comparison, possible age-related maturational differences between comparison groups can be adjusted because the schools serve as their own controls, reducing potential differences between the groups. Furthermore, our study showed a considerable high amount of reports from active travellers and, hence, research is needed to explore recruitment designs that will attract children using different travel modes to school.

## Conclusions

Our results highlight the complexity and challenges in conducting research on AST interventions in a school setting. The children were in general positive about participating in the study and the methods for data collection were considered easy for the participating children to conduct and to blend well with usual school activities. However, explicit group allocation preferences, together with an imbalance in reporting rates between intervention schools and control schools highlight the need to consider alternative research designs regarding large-scale evaluations of AST interventions in a school context. Further, the results indicate that children using active school travel might be more prone to participate and report data than those using non-active travel modes. This invokes the need to explore research designs and recruitment strategies that attract children using all modes of travel.

## Data Availability

The data analyzed in the current study are not publicly available due to ethical restrictions. According to the Swedish regulations (https://etikprovningsmyndigheten.se/, accessed 22 June 2023), permission to use data is only for the purpose for which it has been approved by the Swedish Ethical Review Authority. Data requests can be made to dataskydd@du.se. Point of contact (AP) apl@du.se.

## Abbreviations

PA: Physical activity
AST: Active school travel\
SICTA: Sustainable Innovation for Children Transporting Actively
wASTapp: A web-based survey to assess daily AST
